# Chromosome-level genome assembly of Ceroplastes pseudoceriferus Green, 1935 (Hemiptera: Coccidae)

**DOI:** 10.1038/s41597-025-05477-9

**Published:** 2025-07-05

**Authors:** Yaoguang Qin, Zhennan Wang, Jiangni Li

**Affiliations:** 1 Hebei Key Laboratory of Animal Diversity, College of Life Science, Langfang Normal University, No. 100, Aimin West Road, Langfang, 065000 China; 2 State Key Laboratory for Quality Ensurance and Sustainable Use of Dao-Di Herbs, Institute of Chinese Materia Medica, China Academy of Chinese Medical Sciences, No. 16, Nanxiaojie, Dongzhimennei, Beijing, 100700 China

**Keywords:** Genome, Sequencing

## Abstract

Soft scales (Hemiptera: Coccidae) are significant polyphagous pests and majority of which are invasive species. The 364.14 Mb chromosome-level genome of *Ceroplastes pseudoceriferus* was assembled in this work, with a contig N50 length of 6.16 Mb and scafold N50 length of 21.24 Mb. Approximately 99.89% of assembled sequences were anchored into 18 chromosomes with the assistance of Hi-C reads. Furthermore, approximately 53.98% of the genome was composed of repetitive elements. In total, 10,475 protein-coding genes were predicted, of which 9503 (90.72%) genes were functionally annotated. The BUSCO analysis demonstrated the completeness of the genome annotation is 92.54%. This genome represents first high-quality chromosome level assembly of Coccidae, thereby advancing our knowledge of Coccidae insects and developing effective management strategies that protect crops, forests, and natural ecosystems.

## Background & Summary

Soft scales (Coccidae) is the third largest family within Subfamily Coccoidea behind Diaspididae (armoured scales) and Pseudococcidae (mealybugs)^1–3^. The majority of Coccidae species constitute significant polyphagous pests, posing a threat to economically vital agricultural and horticultural crops, as well as ornamental plants^4^. The inherent polyphagous characteristics and broad host plants of Coccidae species facilitate their widespread invasion globally. Furthermore, Coccidae insects play a pivotal role in ecosystem dynamics through complex interaction with predatory insects, parasitoids and entomopathogens^5^. Understanding the biology, behavior, and ecology of Coccidae insects is crucial for developing effective management strategies that protect crops, forests, and natural ecosystems. Until now, six genomes of Coccidae species are available from GenBank (Table 1), and only one scaffold-level genome has been published as research paper^6^. The scarcity of genomic resources poses a significant obstacle to intensive research endeavors aimed at advancing our knowledge of Coccidae insects.

**Table 1:** Assembly features for genomes of Ceroplastes pseudoceriferus and other Coccidae species.

Organism Name	Assembly Stats Total Sequence Length	Assembly Level	Assembly Stats Total Number of Chromosomes	Assembly Stats Contig N50	Assembly Stats Scaffold N50	Assembly Stats Number of Scaffolds
*Ceroplastes pseudoceriferus*	364,527,212	Chromosome	18	6,164,612	21,238,502	78
*Coccus hesperidum*	405,400,678	Chromosome	7	8,466,229	54,289,485	41
*Toumeyella liriodendri*	536,180,826	Chromosome	17	1,303,606	30,207,406	366
*Ericerus pela*	654,859,808	Scaffold		410,234	1,243,548	2396
*Coccus hesperidum*	484,566,906	Scaffold		399,496	44,350,661	6006
*Parthenolecanium corni*	231,203,063	Contig		14,265,238		
*Ericerus pela*	660,374,735	Contig		660,240		

**Fig. 1:**
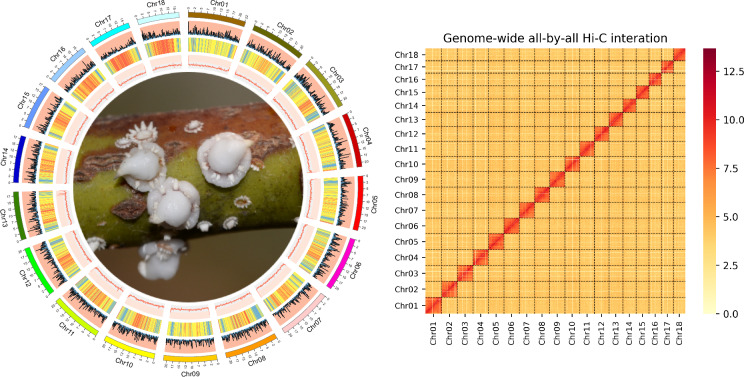
Left: Overview of assembled C. pseudoceriferus genome.The outer layer of coloured blocks is a circular representation of the 18 chromosomes and circos demonstration of gene count (histogram), repeat density (heatmap) and GC content (line) from the outer to the inner circle, respectively. Right: The heatmap represents 18 chromosomes of the C. pseudoceriferus genome.

*Ceroplastes pseudoceriferus*, first described by Green in 1935, is a highly polyphagous species of Coccidae. This species could infest a diverse array of plant species, encompassing 91 genera belonging to 56 plant families^1^. This broad host range underscores potential of *C. pseudoceriferus* as an invasive species. A high-quality reference genome of *C. pseudoceriferus* would be invaluable in enhancing our comprehension of ecological implications of Coccidae and facilitating the development of effective management strategies. In this study, we generated 35.11 Gb of MGI short-read sequencing and 102.21 Gb of Oxford Nanopore Technologies long-read sequencing, 77.20 Gb of high-throughput chromosome conformation capture (Hi-C). The final genome assembly size was 364.52 Mb with an N50 of 6.25 Mb. The karyotype of Ceroplastes pseudoceriferus is characterized by 18 chromosomes at the chromosome level. This conclusion is directly supported by the Hi-C scaffolding results in the genome assembly, where 99.89% of the assembled sequences (364.14 Mb) were anchored into 18 distinct chromosomes with an N50 of 21.24 Mb (Fig. 1).

The assembly achieved an L50 value of 9, indicating that 50% of the total genome length (excluding ambiguous bases) is contained within the 9 longest scaffolds. This demonstrates high continuity in the assembly. The calculated auN value of 20,385,303.53 bp reflects a robust representation of long contiguous sequences, suggesting that the majority of the genome is assembled into large, uninterrupted segments. A total of 60 gaps were identified in the assembly, corresponding to regions of undetermined sequence (represented by runs of ‘N’ bases). This low gap count underscores the assembly’s high completeness. The assembly contains 6,000 ambiguous bases (N), accounting for <0.01% of the total genome length. Such minimal unresolved regions further validate the assembly’s reliability for downstream analyses.

We totally identified 998,369 repeat elements and the total length is 196,755,237 bp, accounting for 53.98% of whole sequence (Table 2). The total length of 23,595 tandem repeats (TR) is 1,374,491 bp, of which length of 10,466 simple sequence repeats (SSRs) is 123,925 bp. The annotation of transposable elements (TE) identified 873,421 repeat elements and the length is 179,535,462 bp, comprising 49.25% of the total. The annotation of non-coding RNAs (ncRNAs) totally resulted in 31 rRNA, 75 small RNA, 109 regulatory and 204 tRNA (Table 3). The gene prediction yielded a total of 10,475 genes, with an average gene length of 20,039 bp, an average CDS length of 1,701 bp, an average exons number per gene at 9, an average exon length of 199 bp, and an average intron length of 2,431 bp (Table 4).

**Table 2:** Repetitive elements sequence statistics of the assembled genome.

Type				Number of elements	Length of sequence (bp)	Percentage of sequence (%)
TEs	Class I	LINE	L2	4,919	746,213	0.20
			Unknown	43,018	7,569,999	2.08
			I-Jockey	3,377	529,766	0.15
			RTE-RTE	3,253	859,132	0.24
			R1	1,414	733,018	0.20
			Other	4,310	459,257	0.13
		LTR	Unknown	42,629	7,267,293	1.99
			Gypsy	8,329	1,639,811	0.45
			Pao	3,007	1,311,568	0.36
			Copia	7,480	2,085,659	0.57
			Other	3,372	385,307	0.11
		SINE	Unknown	8,093	666,156	0.18
			tRNA-Core-RTE	1,795	429,843	0.12
			Other	2,191	621,833	0.17
	Class II	DNA	Kolobok-T2	5,320	428,734	0.12
			TcMar-Tc1	5,396	982,730	0.27
			Unknown	571,953	118,603,002	32.54
			Maverick	6,470	1,382,554	0.38
			hAT-Ac	29,650	4,379,726	1.20
			Crypton-I	6,606	949,425	0.26
			CMC-Chapaev-3	1,962	813,420	0.22
			CMC-EnSpm	2,602	2,788,889	0.77
			TcMar-Fot1	1,893	514,523	0.14
			Academ-1	1,404	469,109	0.13
			TcMar-Mariner	6,415	1,991,663	0.55
			Other	19,541	2,482,897	0.68
		MITE	Unknown	57,951	14,793,825	4.06
		RC	Helitron	19,071	3,650,110	1.00
	Total TEs			873,421	179,535,462	49.25
Tandem Repeats		tandem_repeat		13,129	1,250,566	0.34
		SSR		10,466	123,925	0.03
		Total		23,595	1,374,491	0.38
Simple repeats				2,243	340,996	0.09
Unknown				97,246	15,186,412	4.17
Other				1,759	303,452	0.08
Low complexity				105	14,424	0.00
Total Repeats				998,369	196,755,237	53.98

**Table 3:** Statistics of annotated non-coding RNAs.

Type		Copy Number	Average Length(bp)	Total Length(bp)	Percentage of sequence(%)
rRNA (31)	18S	6	2,669.17	16,015	0.0044
	28S	6	6,343.00	38,058	0.0104
	5.8S	6	158	948	0.0003
	5S	13	116.85	1,519	0.0004
small RNA (75)	snRNA	19	113.11	2,149	0.0006
	miRNA	23	80.65	1,855	0.0005
	spliceosomal	25	140.12	3,503	0.001
	other	8	247	1,976	0.0005
Regulatory	cis-regulatory elements	109	48.61	5,298	0.0015
tRNA	tRNA	204	75.35	15,372	0.0042

**Table 4:** Statistical results of gene structure prediction.

	Gene set	Total number of genes	Average gene length(bp)	Average CDS length(bp)	Average exons number per gene	Average exon length(bp)	Average intron length(bp)
Transcriptome	NGS RNA seq	15,964	30,114.73	3,481.19	9.79	355.63	3,030.40
	PASA	15,637	29,593.23	3,477.10	9.75	356.7	2,985.39
Homoloy	*C. lectularius*	15,795	52,434.27	1,417.84	7.72	183.62	7,589.76
	*A. pisum*	25,118	35,660.83	1,328.27	5.84	227.47	7,094.62
	*N. lugens*	27,001	38,850.77	1,323.46	5.67	233.35	8,033.07
	*H. halys*	20,824	44,447.92	1,351.53	6.63	203.79	7,651.91
	*D. melanogaster*	17,764	28,949.25	1,156.09	5.13	225.37	6,729.98
	GeMoMa	11,353	29,039.92	1,443.02	7.13	202.33	4,500.38
*De novo*	AUGUSTUS	12,124	19,075.14	1,665.97	8.42	197.75	2,344.77
Final	EVM	10,475	20,038.57	1,700.51	8.54	199.03	2,430.89

**Fig. 2:**
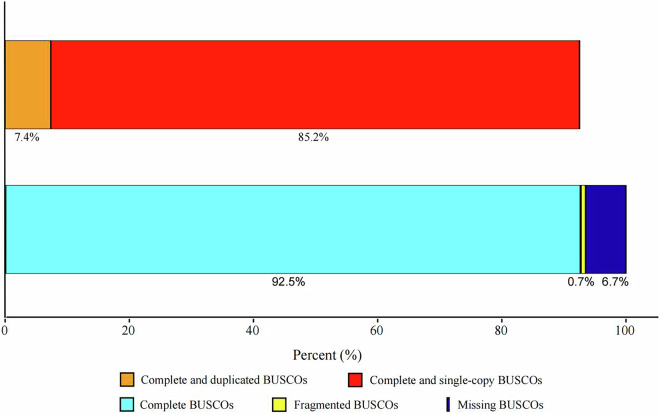
BUSCO evaluation on the genome assembly completeness of the assembled genome.

**Fig. 3:**
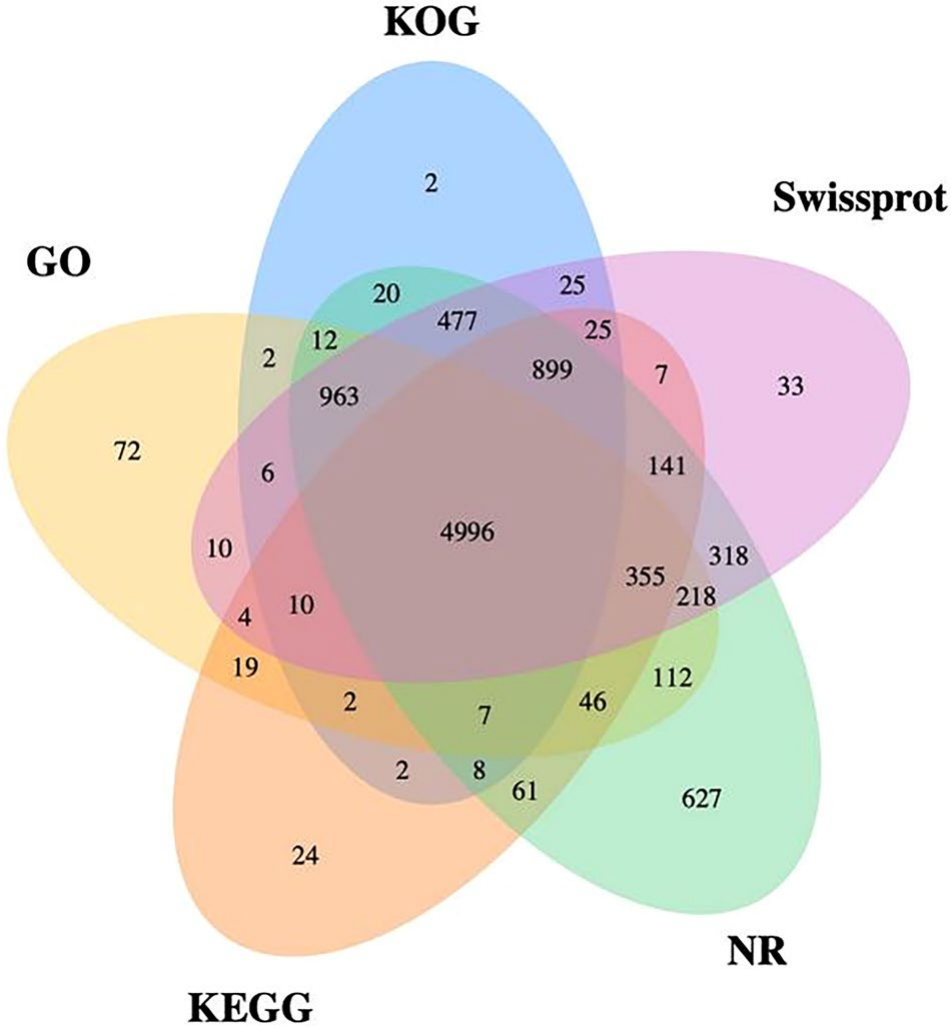
Venn diagram of function annotations from various databases. The Venn diagram displays the overlap and uniqueness of functional gene annotations derived from five databases: SwissProt, Non-Reduntant Protein Database (NR), Kyoto Encyclopedia of Gene and Genomes (KEGG), Eukaryotic Orthologous Groups of protein (KOG) and Gene Ontology (GO).

The completeness of the predicted genes was assessed using Benchmarking Universal Single-Copy Orthologs (BUSCO)^7^, resulting in a high score of 92.54% (n = 1,265) (Fig. 2). This encompassed 85.15% (1164) single-copy, 7.39% (101) duplicated, 0.73% (10) fragmented, and 6.73% (92) missing BUSCOs, indicating a high integrity of gene prediction. Functional annotation indicated that a total of 90.72% (9503) of genes were annotated to five public databases including SwissProt^8^, the NCBI non-reduntant protein database (NR), Kyoto Encyclopedia of Gene and Genomes (KEGG)^9^, Eukaryotic Orthologous Groups of protein (KOG)^10^ and Gene Ontology (GO)^11^ (Fig. 3).

## Method

### Sample collection, library construction and sequencing

First-instar larva of *C. pseudoceriferus* were collected from a single Camphor tree in Kunming, Yunnan Province, China (25°02′11″N, 102°42′31″E). Specimens were flash-frozen in liquid nitrogen and stored at −80 °C. Thousands of whole-body samples were used for DNA and RNA extraction to ensure sufficient biological material for multi-omics sequencing, including MGI short-read sequencing, Nanopore long-read sequencing, Hi-C sequencing and RNA sequencing. Genomic DNA (gDNA) was extracted using a QIAGEN® Genomic DNA Kit (Cat#13323). Total RNA was extracted using TRIzol® (TIANGEN) with DNase I treatment. All DNA and RNA extractions were conducted by Grandomics Biosciences (Wuhan, China) following manufacturer recommended protocols, meeting the quality specifications for library preparation and sequencing requirements.

For MGI sequencing, gDNA was randomly fragmented and libraries were prepared using standard protocols. After quality checks, the libraries were sequenced on the MGI DNBSEQ-T7RS platform, producing 35.11 Gb of raw data with 100 × average coverage.

For Nanopore sequencing^12^, size-selected DNA fragments were processed using the PippinHT system (Sage Science). Following end-repair and adapter ligation (SQK-LSK114 kit), library concentrations were quantified with a Qubit fluorometer. The prepared library was sequenced on a PromethION platform (Oxford Nanopore Technologies), yielding 102.21 Gb of long-read data at 287 × coverage for genome assembly.

For Hi-C sequencing, whole body samples were formaldehyde-fixed (2%) to crosslink DNA-protein complexes. Libraries were constructed through chromatin digestion, proximity ligation, and biotin pulldown following the protocol^13^. Sequencing on the MGISEQ-2000 platform generated 77.20 Gb of 150 bp paired-end reads (217 × coverage), enabling 3D chromatin architecture analysis.

For RNA sequencing, the Poly A mRNA was enriched via Dynabeads, fragmented, and reverse-transcribed using MGIEasy RNA Library Prep Kit V3.1 (Cat# 1000005276, MGI). Libraries were circularized and sequenced on DNBSEQ-T7RS, generating 13.45 Gb data for gene annotation.

### Genome assembly

The raw Nanopore reads underwent *de novo* genome assembly using NextDenovo (v2.5.2)^14^ with default parameters. Four iterative correction rounds of Nextpolish (v1.2.4)^15^ polishing were subsequently applied using MGI short reads to enhance base accuracy. Assembly quality was verified through mapping all MGI reads to the assembled genome using BWA (Burrows-Wheeler Aligner, v0.7.12-r1039)^16^ and coverage assessment using Winnowmap2^17^ with parameters of “-x map-ont”. Base-level accuracy was calculated using samtools (v1.4)^18^ and BCFtools (v1.8.0)^19^ with default parameters. Mitochondrial sequence exclusion was performed via BLAST (v2.9)^20^ alignment against the NT database, followed by contaminant removal.

### Chromosome anchoring

The 370 million paired-end reads underwent quality control through the standardized Hi-C data processing workflow^21^. Low-quality sequences (quality scores <20), adaptor sequences and sequences shorter than 30 bp were filtered out using fastp (v0.21.0)^22^. Clean reads were mapped using bowtie2 (v2.3.2)^23^ (-end-to-end–very-sensitive -L 30). HiC-Pro (v2.8.1)^21^ retained valid chromatin contacts while filtering invalid read pairs, including dangling-end, self-cycle, re-ligation, and dumped products. Scaffolds were organized into chromosomes using LACHESIS^24^, with key parameters CLUSTER_MIN_RE_SITES = 100, CLUSTER_MAX_LINK_DENSITY = 2.5, CLUSTER NONINFORMATIVE RATIO = 1.4, ORDER MIN N RES IN TRUNK = 60, ORDER MIN N RES IN SHREDS = 60. Final chromosomal orientations were manually verified and corrected based on chromatin interaction patterns.

### Repeat annotation

Tandem repeats were systematically annotated through complementary approaches: simple sequence repeats (SSRs) were detected using GMATA (v2.2)^25^ with default parameters, while genome-wide tandem repeats were identified via Tandem Repeats Finder (TRF V4.07b)^26^ using sensitivity thresholds of “2 7 7 80 10 50 500 -f -d -h -r”. Transposable elements (TE) annotation was combinig *ab initio* and homology-based methods. *ab initio* repeat library was predicted using MITE-hunter^27^ with parameters of “-n 20 -P 0.2 -c 3” and RepeatModeler version open-2.0.4^28^ with parameters of “-engine wublast”, in which long terminal repeats (LTRs) were characterized using LTR_FINDER^29^, *LTRharvest*^30^ and LTR_retriever^31^. The obtained library underwent cross referencing against TEclass Repbase^32^ (http://www.girinst.org/repbase) for TE classification. Genome-wide TE masking was subsequently performed using RepeatMasker^33^ which simultaneously screened sequences against both the de novo library and RepBase reference. Overlapping TE annotations were resolved through BEDTools-based merging followed by manual curation to validate complex repeat architectures.

### Annotation of non-coding RNAs (ncRNAs)

Non-coding RNA identification employed complementary database alignment and model-based prediction approaches. Transfer RNA genes were systematically predicted through tRNAscan-SE (v2.0)^34^ using eukaryotic configuration parameters. For comprehensive detection of small regulatory RNAs (miRNAs, snRNAs, snoRNAs) and ribosomal RNAs, a dual-strategy framework was implemented: Infernal (v1.1.2)^35^ facilitated covariance model searches against the Rfam Database^36^, while RNAmmer (v1.2)^37^ provided specialized prediction of rRNA subunits through hidden Markov model profiling. This integrated annotation pipeline ensured cross-validated identification of non-coding RNA elements across multiple functional categories.

### Gene prediction

Gene annotation integrated three complementary methodologies applied to the repeat-masked genome. Homology-based predictions were generated through GeMoMa (v1.6.1)^38^, which mapped conserved protein sequences from five phylogenetically relevant species (*Acyrthosiphon pisum*^39^, *Cimex lectularius*^40^, *Drosophila melanogaster*^41^, *Halyomorpha halys*^42^ and *Nilaparvata lugens*^43^) onto the assembly. Concurrently, transcriptomic evidence was incorporated through RNA-seq alignment using STAR-2.7.3a^44^, followed by transcript assembly with StringTie2^45^ and ORF prediction via PASA (v2.3.3)^46^. For the *ab initio* prediction, RNA-seq-derived training sets processed through StringTie and PASA informed Augustus (v3.3.1)^47^ predictions. The three prediction streams were reconciled through EVidenceModeler (EVM)^46^, creating a unified gene set subsequently refined through TransposonPSI^48^ filtration to remove transposon associated genes. Structural annotation completeness was enhanced through PASA-based identification of untranslated regions (UTRs) and alternative splicing variants, with canonical isoforms selected based on maximum ORF length. Final gene models retained only protein-coding sequences validated through evolutionary conservation, transcriptional evidence, and *ab initio* support, ensuring both structural accuracy and biological relevance.

### Functional annotation of gene

Gene functional characterization was conducted through systematic interrogation of five biological databases using complementary bioinformatics approaches. Protein domain architecture and Gene Ontology (GO) term assignments were determined via InterProScan^49^ analysis with default parameterization. Parallel BLASTp searches (E-value threshold ≤ 1e–5) against four major databases - SwissProt (manually curated proteins), NR (non-redundant sequences), KEGG (metabolic pathways), and KOG (eukaryotic ortholog clusters) - were performed on the EvidenceModeler-derived proteome, retaining only top-scoring alignments for each query sequence. The consensus functional annotations derived from these five independent evidence streams (InterProScan domain predictions and four BLAST-based database matches) were subsequently integrated through hierarchical evidence weighting, prioritizing experimentally validated annotations from SwissProt while incorporating complementary functional predictions from other resources. This multi-tiered annotation framework ensured comprehensive functional insights spanning molecular interactions, biological processes, and evolutionary relationships.

## Data Records

The raw sequence data reported in this paper have been deposited in the Genome Sequence Archive^50^ in National Genomics Data Center^51^, China National Center for Bioinformation/Beijing Institute of Genomics, Chinese Academy of Sciences (GSA: CRA019415^52^) that are publicly accessible at https://ngdc.cncb.ac.cn/gsa. The whole genome sequence data and the annotation files for genome, ncRNA and repeat content are available from the Figshare repository^53–56^. The whole genome sequence are also deposited in the National Center for Biotechnology Information (NCBI) GenBank, with accession number GCA_050872025.1^57^, under BioProject PRJNA1273070.

## Technical Validation

High mapping quality were confirmed in our results. After assembling ONT reads, BWA was used to align MGI short-reads with the assembled genome, resulted in mapping rate of 98.70%. The coverage of the genome by MGI short-reads is 99.72%. Winnowmap2 was used to align ONT reads with the assembled genome, resulted in mapping rate of 98.65%, and achieving a genome coverage of 99.99%. The combination of parthenogenetic tendencies in Coccidae, single-host sampling, and high assembly quality strongly suggests that these first-instar larvae had low genetic diversity, making them suitable for producing a coherent reference genome.

## Data Availability

All software used in this study is in the public domain and its parameters are clearly described in the methodology. If the detailed parameters of the software are not mentioned, the default parameters are used as recommended by the developer. No custom code or scripts were used for the curation and validation of the dataset.

## References

[1] Morales, M. G. *et al*. ScaleNet: a literature-based model of scale insect biology and systematics. *Database* **2016**, bav118 (2016). 26861659 10.1093/database/bav118 PMC4747323

[2] Wang, X. B. *et al*. DNA barcoding of common soft scales (Hemiptera: Coccoidea: Coccidae) in China. *Bull. Entomol. Res.* **105**(5), 545–554, 10.1017/S0007485315000413 (2015). 25989705 10.1017/S0007485315000413

[3] Choi, J., Soysouvanh, P., Lee, S. & Hong, K. J. Review of the family Coccidae (Hemiptera: Coccomorpha) in Laos. *Zootaxa* **4460**(1), 1–62, 10.11646/zootaxa (2018). 30314105 10.11646/zootaxa.4460.1.1

[4] Bendov, Y. A systematic catalogue of the soft scale insects of the world (Homoptera: Coccoidea: Coccidae) with data on geographical distribution, host plants, biology and economic importance. *Annals of the Entomological Society of America* **86**(6), 841–841(1) (1993).

[5] Becchimanzi, A., Nicoletti, R., Di Lelio, I. & Russo, E. Immune Gene Repertoire of Soft Scale Insects (Hemiptera: Coccidae). *Int. J. Mol. Sci.* **25**, 4922, 10.3390/ijms25094922 (2024). 38732132 10.3390/ijms25094922 PMC11084805

[6] Yang, P. *et al*. Genome sequence of the Chinese white wax scale insect *Ericerus pela*: the first draft genome for the Coccidae family of scale insects. *Gigascience.* **8**(9), giz113, 10.1093/gigascience/giz113 (2019). 31518402 10.1093/gigascience/giz113 PMC6743827

[7] Simão, F. A. *et al*. BUSCO: assessing genome assembly and annotation completeness with single-copy orthologs. *Bioinformatics.* **31**(19), 3210–3212, 10.1093/bioinformatics/btv351 (2015). 26059717 10.1093/bioinformatics/btv351

[8] Bairoch, A. & Apweiler, R. The SWISS-PROT protein sequence data bank and its supplement TrEMBL in 1999. *Nucleic Acids Res.* **27**, 49–54, 10.1093/nar/27.1.49 (1999). 9847139 10.1093/nar/27.1.49 PMC148094

[9] Ogata, H. *et al*. KEGG: Kyoto Encyclopedia of Genes and Genomes. *Nucleic Acids Res.* **27**, 29–34, 10.1093/nar/27.1.29 (1999). 9847135 10.1093/nar/27.1.29 PMC148090

[10] Galperin, M. Y. *et al*. Expanded microbial genome coverage and improved protein family annotation in the COG database. *Nucleic Acids Res.* **43**, D261–D269, 10.1093/nar/gku1223 (2015). 25428365 10.1093/nar/gku1223 PMC4383993

[11] Ashburner, M. *et al*. Gene Ontology: tool for the unification of biology. *Nat. Genet.* **25**, 25–29, 10.1038/75556 (2000). 10802651 10.1038/75556 PMC3037419

[12] Shang, L. *et al*. A super pan-genomic landscape of rice. *Cell Res.* **32**, 878–896, 10.1038/s41422-022-00685-z (2022). 35821092 10.1038/s41422-022-00685-z PMC9525306

[13] Rao, S. S. P. *et al*. A 3D Map of the Human Genome at Kilobase Resolution Reveals Principles of Chromatin Looping. *Cell* **159**, 1665–1680, 10.1016/j.cell.2014.11.021 (2014). 25497547 10.1016/j.cell.2014.11.021 PMC5635824

[14] Hu, J. *et al*. NextDenovo: an efficient error correction and accurate assembly tool for noisy long reads. *Genome Biol.* **25**, 107, 10.1186/s13059-024-03252-4 (2024). 38671502 10.1186/s13059-024-03252-4 PMC11046930

[15] Hu, J. *et al*. NextPolish: a fast and efficient genome polishing tool for long-read assembly. *Bioinformatics* **36**, 2253–2255, 10.1093/bioinformatics/btz891 (2020). 31778144 10.1093/bioinformatics/btz891

[16] Li, H. & Durbin, R. Fast and accurate long-read alignment with Burrows–Wheeler transform. *Bioinformatics* **26**, 589–595, 10.1093/bioinformatics/btp698 (2010). 20080505 10.1093/bioinformatics/btp698 PMC2828108

[17] Jain, C. *et al*. Long-read mapping to repetitive reference sequences using Winnowmap2. *Nat Methods* **19**, 705–710, 10.1038/s41592-022-01457-8 (2022). 35365778 10.1038/s41592-022-01457-8 PMC10510034

[18] Li, H. *et al*. The Sequence Alignment/Map format and SAMtools. *Bioinformatics* **25**, 2078–2079, 10.1093/bioinformatics/btp352 (2009). 19505943 10.1093/bioinformatics/btp352 PMC2723002

[19] Danecek, P. & McCarthy, S. A. BCFtools/csq: haplotype-aware variant consequences. *Bioinformatics* **33**, 2037–2039, 10.1093/bioinformatics/btx100 (2017). 28205675 10.1093/bioinformatics/btx100 PMC5870570

[20] Chaisson, M. J. & Tesler, G. Mapping single molecule sequencing reads using basic local alignment with successive refinement (BLASR): application and theory. *BMC Bioinformatics* **13**, 238, 10.1186/1471-2105-13-238 (2012). 22988817 10.1186/1471-2105-13-238 PMC3572422

[21] Servant, N. *et al*. HiC-Pro: an optimized and flexible pipeline for Hi-C data processing. *Genome Biol.* **16**, 259, 10.1186/s13059-015-0831-x (2015). 26619908 10.1186/s13059-015-0831-x PMC4665391

[22] Chen, S. *et al*. fastp: an ultra-fast all-in-one FASTQ preprocessor. *Bioinformatics* **34**, i884–i890, 10.1093/bioinformatics/bty560 (2018). 30423086 10.1093/bioinformatics/bty560 PMC6129281

[23] Langmead, B. & Salzberg, S. L. Fast gapped-read alignment with Bowtie 2. *Nat. Methods* **9**, 357–359, 10.1038/nmeth.1923 (2012). 22388286 10.1038/nmeth.1923 PMC3322381

[24] Burton, J. N. *et al*. Chromosome-scale scaffolding of *de novo* genome assemblies based on chromatin interactions. *Nat. Biotechnol.* **31**, 1119–1125, 10.1038/nbt.2727 (2013). 24185095 10.1038/nbt.2727 PMC4117202

[25] Wang, X. & Wang, L. GMATA: An Integrated Software Package for Genome-Scale SSR Mining, Marker Development and Viewing. *Front. Plant Sci.* **7**, 1350, 10.3389/fpls.2016.01350 (2016). 27679641 10.3389/fpls.2016.01350 PMC5020087

[26] Benson, G. Tandem repeats finder: a program to analyze DNA sequences. *Nucleic Acids Res.* **27**, 573–580, 10.1093/nar/27.2.573 (1999). 9862982 10.1093/nar/27.2.573 PMC148217

[27] Han, Y. & Wessler, S. R. MITE-Hunter: a program for discovering miniature inverted-repeat transposable elements from genomic sequences. *Nucleic Acids Res.* **38**, e199–e199, 10.1093/nar/gkq862 (2010). 20880995 10.1093/nar/gkq862 PMC3001096

[28] Flynn, J. M. *et al*. RepeatModeler2 for automated genomic discovery of transposable element families. *Proceedings of the National Academy of Sciences* **117**, 9451–9457, 10.1073/pnas.1921046117 (2020). 10.1073/pnas.1921046117 PMC7196820 32300014

[29] Xu, Z. & Wang, H. LTR_FINDER: an efficient tool for the prediction of full-length LTR retrotransposons. *Nucleic Acids Res.* **35**, W265–W268, 10.1093/nar/gkm286 (2007). 17485477 10.1093/nar/gkm286 PMC1933203

[30] Ellinghaus, D., Kurtz, S. & Willhoef, U. LTRharvest, an efficient and flexible software for *de novo* detection of LTR retrotransposons. *BMC Bioinformatics* **9**, 18, 10.1186/1471-2105-9-18 (2008). 18194517 10.1186/1471-2105-9-18 PMC2253517

[31] Ou, S. & Jiang, N. LTR_retriever: A Highly Accurate and Sensitive Program for Identification of Long Terminal Repeat Retrotransposons. *Plant Physiol.* **176**, 1410–1422, 10.1104/pp.17.01310 (2018). 29233850 10.1104/pp.17.01310 PMC5813529

[32] Abrusán, G., Grundmann, N., DeMester, L. & Makalowski, W. TEclass—a tool for automated classification of unknown eukaryotic transposable elements. *Bioinformatics* **25**, 1329–1330, 10.1093/bioinformatics/btp084 (2009). 19349283 10.1093/bioinformatics/btp084

[33] Bedell, J. A., Korf, I. & Gish, W. MaskerAid: a performance enhancement to RepeatMasker. *Bioinformatics* **16**, 1040–1041, 10.1093/bioinformatics/16.11.1040 (2000). 11159316 10.1093/bioinformatics/16.11.1040

[34] Chan, P. P. *et al*. tRNAscan-SE 2.0: improved detection and functional classification of transfer RNA genes. *Nucleic Acids Res.* **49**, 9077–9096, 10.1093/nar/gkab688 (2021). 34417604 10.1093/nar/gkab688 PMC8450103

[35] Nawrocki, E. P. & Eddy, S. R. Infernal 1.1: 100-fold faster RNA homology searches. *Bioinformatics* **29**, 2933–2935, 10.1093/bioinformatics/btt509 (2013). 24008419 10.1093/bioinformatics/btt509 PMC3810854

[36] Grifths-Jones, S. *et al*. Rfam: annotating non-coding RNAs in complete genomes. *Nucleic Acids Res.* **33**, D121–D124, 10.1093/nar/gki081 (2005). 15608160 10.1093/nar/gki081 PMC540035

[37] Lagesen, K. *et al*. RNAmmer: consistent and rapid annotation of ribosomal RNA genes. *Nucleic Acids Res.* **35**, 3100–3108, 10.1093/nar/gkm160 (2007). 17452365 10.1093/nar/gkm160 PMC1888812

[38] Keilwagen, J. *et al*. Using intron position conservation for homology-based gene prediction. *Nucleic Acids Res.* **44**, e89–e89, 10.1093/nar/gkw092 (2016). 26893356 10.1093/nar/gkw092 PMC4872089

[39] International Aphid Genomics Consortium. Genome sequence of the pea aphid *Acyrthosiphon pisum*. *PLoS biology.* **8**(2), e1000313, 10.1371/journal.pbio.1000313 (2010). 20186266 10.1371/journal.pbio.1000313 PMC2826372

[40] Rosenfeld, J. *et al*. Genome assembly and geospatial phylogenomics of the bed bug *Cimex lectularius*. *Nat. Commun.* **7**, 10164, 10.1038/ncomms10164 (2016). 26836631 10.1038/ncomms10164 PMC4740774

[41] Adams, M. D. *et al*. The genome sequence of *Drosophila melanogaster*. *Science.* **287**(5461), 2185–2195, 10.1126/science.287.5461.2185 (2000). 10731132 10.1126/science.287.5461.2185

[42] Sparks, M. E. *et al*. Brown marmorated stink bug, *Halyomorpha halys* (Stål), genome: putative underpinnings of polyphagy, insecticide resistance potential and biology of a top worldwide pest. *BMC Genomics.* **21**(1), 227, 10.1186/s12864-020-6510-7 (2020). 32171258 10.1186/s12864-020-6510-7 PMC7071726

[43] Ye, Y. X. *et al*. Chromosome-level assembly of the brown planthopper genome with a characterized Y chromosome. *Mol. Ecol. Resour.* **21**(4), 1287–1298, 10.1111/1755-0998.13328 (2021). 33460519 10.1111/1755-0998.13328

[44] Dobin, A. *et al*. STAR: ultrafast universal RNA-seq aligner. *Bioinformatics* **29**, 15–21, 10.1093/bioinformatics/bts635 (2013). 23104886 10.1093/bioinformatics/bts635 PMC3530905

[45] Kovaka, S. *et al*. Transcriptome assembly from long-read RNA-seq alignments with StringTie2. *Genome Bio.* **20**, 278, 10.1186/s13059-019-1910-1 (2019). 31842956 10.1186/s13059-019-1910-1 PMC6912988

[46] Haas, B. J. *et al*. Automated eukaryotic gene structure annotation using EVidenceModeler and the Program to Assemble Spliced Alignments. *Genome Biol.* **9**, R7, 10.1186/gb-2008-9-1-r7 (2008). 18190707 10.1186/gb-2008-9-1-r7 PMC2395244

[47] Stanke, M. *et al*. Using native and syntenically mapped cDNA alignments to improve *de novo* gene finding. *Bioinformatics* **24**, 637–644, 10.1093/bioinformatics/btn013 (2008). 18218656 10.1093/bioinformatics/btn013

[48] Urasaki, N. *et al*. Draft genome sequence of bitter gourd (*Momordica charantia*), a vegetable and medicinal plant in tropical and subtropical regions. *DNA Res.* **24**(1), 51–58, 10.1093/dnares/dsw047 (2017). 28028039 10.1093/dnares/dsw047 PMC5381343

[49] Zdobnov, E. M. & Apweiler, R. InterProScan–an integration platform for the signature-recognition methods in InterPro. *Bioinformatics* **17**(9), 847–848, 10.1093/bioinformatics/17.9.847 (2001). 11590104 10.1093/bioinformatics/17.9.847

[50] The Genome Sequence Archive Family. Toward Explosive Data Growth and Diverse Data Types. *Genomics, Proteomics & Bioinformatics* **19**(4), 578–583, 10.1016/j.gpb.2021.08.001 (2021). 10.1016/j.gpb.2021.08.001 PMC9039563 34400360

[51] Database Resources of the National Genomics Data Center. China National Center for Bioinformation in 2022. *Nucleic Acids Res.* **50**(D1), D27–D38, 10.1093/nar/gkab951 (2022). 34718731 10.1093/nar/gkab951 PMC8728233

[52] *NGDC Genome Sequence Archive.* https://ngdc.cncb.ac.cn/gsa/browse/CRA019415 (2025).

[53] Qin, Y. G. *Ceroplastes pseudoceriferus* genome. *Figshare*10.6084/m9.figshare.27193908.v1 (2024).

[54] Qin, Y. G. *Ceroplastes pseudoceriferus* genome annotation. *Figshare*10.6084/m9.figshare.27193896.v1 (2024).

[55] Qin, Y. G. *Ceroplastes pseudoceriferus* noncoding annotation. *Figshare*10.6084/m9.figshare.27195201.v1 (2024).

[56] Qin, Y. G. *Ceroplastes pseudoceriferus* repeat content annotation. *Figshare*10.6084/m9.figshare.27193911.v1 (2024).

[57] *NCBI GenBank* https://identifiers.org/ncbi/insdc.gca:GCA_050872025.1 (2025).

